# Stroke Event Rates and the Optimal Antithrombotic Choice of Patients With Paroxysmal Atrial Fibrillation: A Systematic Review and Meta-Analysis of Randomized Controlled Trials

**DOI:** 10.1097/MD.0000000000002364

**Published:** 2015-12-31

**Authors:** Yicong Chen, Yuhui Zhao, Ge Dang, Fubing Ouyang, Xinran Chen, Jinsheng Zeng

**Affiliations:** From the Department of Neurology and Stroke Center, The First Affiliated Hospital, Sun Yat-Sen University, Guangzhou, China.

## Abstract

The risks of stroke or systemic embolism and major bleeding are considered similar between paroxysmal and sustained atrial fibrillation (AF), and warfarin has demonstrated superior efficacy to aspirin, irrespective of the AF type. However, with the advent of novel oral anticoagulants (NOACs) and antiplatelet agents, the optimal antithrombotic prophylaxis for paroxysmal AF remains unclear.

We searched Medline, Embase, CENTRAL, and China Biology Medicine up to October week 1, 2015. Randomized controlled trials of AF patients assigned to NOACs, warfarin, or antiplatelets, with reports of outcomes stratified by the AF type, were included. A fixed-effects model was used if no statistically significant heterogeneity was indicated; otherwise, a random-effects model was used.

Six studies of 69,990 nonvalvular AF patients with ≥1 risk factor for stroke were included. Postantithrombotic treatment, paroxysmal AF patients showed lower risks of stroke (risk ratio [RR], 0.72; 95% confidence interval [CI], 0.59–0.87), stroke or systemic embolism (RR, 0.74; 95% CI, 0.63–0.86), and all-cause mortality (RR, 0.75; 95% CI, 0.67–0.83), while the major bleeding risk was comparable (RR, 0.96; 95% CI, 0.85–1.08). We were unable to detect the superiority of anticoagulation over antiplatelets for paroxysmal AF (RR, 0.72; 95% CI, 0.43–1.23), while it was more effective than antiplatelets for sustained AF (RR, 0.42; 95% CI, 0.33–0.54). NOACs showed superior efficacy over warfarin and trended to show reduced major bleeding irrespective of the AF type.

The AF type is a predictor for thromboembolism, and might be helpful in stroke risk stratification model in combination with other risk factors. With the appearance of novel anticoagulant and antiplatelet agents, the best antithrombotic choice for paroxysmal AF needs further exploration.

## Abstract 2

Supplemental Digital Content is available in the text

## INTRODUCTION

Atrial fibrillation (AF) is associated with 2- to 7-fold increased risks of stroke^1–5^ and higher occurrence of non-central nervous system (non-CNS) systemic embolism.^5^ The correlation between AF and stroke, particularly paroxysmal AF, defined as recurrent AF that terminates spontaneously and lasts up to 7 days, has drawn much attention in recent years. Covert paroxysmal AF has been proposed as a potential cause of embolic stroke of undetermined source (ESUS),^6^ and novel electrocardiogram (ECG) monitoring techniques with 30-day event-triggered recorders^7^ and insertable cardiac monitors^8,9^ have found paroxysmal AF to be associated with cryptogenic ischemic stroke.^7,8^ The AF type is generally considered irrelevant to the stroke risk,^5,10,11^ and the distinction between paroxysmal AF and persistent AF has not been used to guide the choice of stroke prophylaxis; however, increasing studies have suggested that paroxysmal AF carries a lower risk of stroke compared with sustained (persistent or permanent) AF.^12–18^ Whether thromboembolic risk varies by AF type remains uncertain.^11,13,15–21^ The reported relative stroke risks between paroxysmal and sustained AF may be confounded by the treatment of differential anticoagulant use in patients with paroxysmal and sustained AF in some studies.^18,20–23^ Therefore, comparing the risk of thromboembolism between different AF types by performing a pooled analysis according to antithrombotic treatment assignment is needed.

Warfarin is considered more efficacious than aspirin for stroke prevention in AF^10,24,25,45^; thus, anticoagulation prophylaxis is recommended for at-risk patients with paroxysmal or sustained AF.^5,10,26^ However, few studies have specifically evaluated the efficacy and safety of anticoagulant versus antiplatelet agents for paroxysmal AF, and the choice of thromboembolic prophylaxis for paroxysmal AF has become more diversified with the emergence of novel antiplatelet and anticoagulant agents. Novel oral anticoagulants (NOACs) have shown a favorable risk–benefit profile for AF, with reductions in stroke or systemic embolism and similar major bleeding risk as for dose-adjusted warfarin^27–29^; however, whether their advantages extend to both AF types is unknown.

Accordingly, we conducted this meta-analysis to assess the differences in thromboembolism and bleeding risk between paroxysmal and sustained AF patients according to the antithrombotic therapy used, and to detect whether there was a difference in the treatment effect between anticoagulation versus antiplatelets and NOACs versus warfarin in such patients.

## METHODS

### Data Sources and Searches

The Preferred Reporting Items for Systematic reviews and Meta-Analyses (PRISMA) guidelines were followed. We firstly identified published studies that compared the efficacy and safety outcomes by AF type in patients randomized to antithrombotic therapies through systematically searching Medline (Ovid, 1946 to September 2014), Embase (Ovid, 1974 to September 2014), Cochrane Central Register of Controlled Trials (CENTRAL) (Ovid, September 2014), and China Biology Medicine disc (SinoMed, 1978 to September 2014). We updated the search up to October week 1, 2015 for any additional eligible studies. Medical subject headings (MeSH) and the terms “atrial fibrillation,” “AF,” “stroke,” “brain infarction,” “brain vascular accident,” “cerebrovascular accident,” and “embolism” were used and the randomized controlled trials (RCT) filters for Medline and Embase in Ovid Expert Search were applied (see TEXT 1, Supplemental Content, which illustrates the search strategy). No language restriction was used. Additionally, we manually reviewed the reference lists of related reviews, editorials, and studies identified after title and abstract screening for potential relevant studies. This cross-checking was repeated until no further studies were identified.

### Study Selection

Two reviewers (YC and YZ) performed the study selection independently, with disagreements solved through discussion or by judgment of a third reviewer (JZ). The study inclusion criteria were: phase III RCTs comparing the efficacy and safety of NOACs, warfarin, or antiplatelet therapy in AF patients; studies including secondary analyses stratified by AF types with the endpoints of stroke, composite of stroke or non-CNS systemic embolism, all-cause mortality, or major bleeding; and ≥1-year follow-up.

### Data Extraction and Quality Assessment

Data on the included studies (publication year, inclusion criteria, follow-up period, studied drugs), population characteristics (age, sex, comorbidities, medication use at entry), treatment (therapeutic indication, drug, dosage), and outcomes were extracted using a standardized data extraction form. For trials reported >1 publications, we extracted data from the most complete one and used the others to supplement the data.

Outcome information was stratified by paroxysmal and sustained AF. The primary efficacy outcome was stroke or non-CNS systemic embolism. Secondary efficacy outcomes included stroke (ischemic, hemorrhagic, unspecified) and all-cause mortality. The primary safety outcome was major bleeding, defined according to the International Society on Thrombosis and Hemostasis criteria as clinically overt bleeding accompanied by a fall in the hemoglobin level of ≥2 g/dL, transfusion of ≥2 units of whole or packed red blood cells, occurring in a critical site, or leading to death.^30^

Among the trials included, AF was mainly diagnosed by local investigators at the time of enrollment according to ECG and the previous medical history. Paroxysmal AF was defined as recurrent AF self-terminating within 7 days; when persisting beyond 7 days or terminated upon pharmacological therapy or electrical cardioversion, it was considered persistent. Permanent AF referred to long-standing AF with no evidence of sinus rhythm for several months prior to randomization (see TEXT 2, Supplemental Content, which illustrates the definitions and classifications of AF type).^10^ Because persistent AF has a tendency to convert into permanent AF, and since both sustain beyond 7 days, we combined these 2 groups into sustained AF.^10^

Study quality assessment was performed following a validated scale for RCTs recommended by the Cochrane Collaboration,^31^ by evaluating the random sequence generation, allocation concealment, blinding of participant and personnel, blinding of outcome assessment, incomplete outcome data, selective reporting, and other biases. Each item was evaluated as high, low, or unclear risk. A study was classified as low risk when every item was considered low risk, and as high or unclear risk if 1 or more items were evaluated as being high or unclear risk, respectively. Discrepancies about the quality assessment were resolved by consensus.

### Data Synthesis and Analysis

The population baseline characteristics according to the AF type were analyzed using Pearson's chi-squared test for categorical variables in SPSS 16.0 for windows (SPSS Inc., Chicago, IL).

Pooled risk ratios (RRs) and 95% confidence intervals (CIs) were calculated for each outcome. Subgroup analysis was conducted according to anticoagulant (NOACs and warfarin) and antiplatelet treatment to assess the comparative risks of stroke or systemic embolism and major bleeding between paroxysmal and sustained AF. The efficacy and safety outcomes were also compared for anticoagulation versus antiplatelet treatment; and for NOACs versus warfarin. Heterogeneity was assessed by comparing the inclusion criteria and the design and conduct differences of the trials. Heterogeneity across studies was assessed by the Q test and I^2^ index, which measures the proportion of total variability attributable to between-studies differences rather than sampling error. We synthesized and compared outcomes by a fixed-effects model (Mantel–Haenszel method) if no statistically significant heterogeneity was indicated (*P* > 0.10 with I^2^ < 50%); otherwise, a random-effects model was used. Because the Randomized Evaluation of Long-Term Anticoagulation Therapy (RE-LY) trial provided only RRs with 95% CIs rather than event numbers for each outcome by AF type,^32^ in the comparison of NOACs and warfarin, we transformed the data into ln(RR) and standard error of ln(RR) (SE[lnRR]) and performed data synthesis using the Inverse-Variance method. SE(lnRR) was calculated as [ln(95% CI[upper limit]) − lnRR]/1.96. *P* values ≤0.05 were considered significant.

All analyses were performed with Review Manager, version 5.2 (Copenhagen: The Nordic Cochrane Centre, The Cochrane Collaboration, 2012).

## RESULTS

**FIGURE 1:**
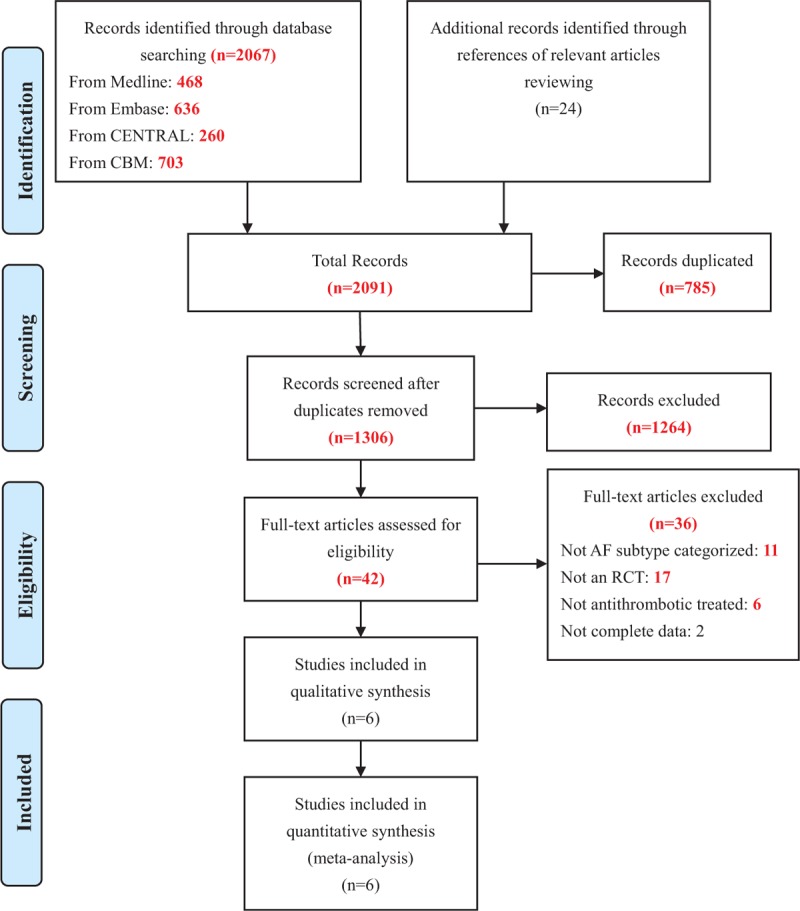
Flow diagram of study selection.

We identified a total of 2091 studies through database and manual searches, of which the full text of 42 were evaluated based on our inclusion criteria; eventually, 6 were eligible for inclusion (Fig. 1).^13,16,17,21,22,32^

### Study Characteristics and Study Quality

Six phase III RCTs, including 69,990 participants, comparing the efficacy and safety of NOACs, warfarin, or antiplatelet therapy in nonvalvular AF patients with ≥1 risk factor for stroke, which included secondary analyses of the rates of stroke or systemic embolism and major bleeding stratified by AF type, were identified (Table 1).^13,16,17,21,22,32^ Specifically, the Rivaroxaban Once Daily Oral Direct Factor Xa Inhibition Compared with Vitamin K Antagonism for Prevention of Stroke and Embolism (ROCKET-AF) trial enrolled a high risk population with ≥2 risk factors.^17^ Four trials involved comparisons of NOACs (apixaban, rivaroxaban, dabigatran, and ximelagatran) versus warfarin.^13,16,17,32^ One study examined the effects of apixaban and aspirin in patients who failed or were unsuitable for vitamin K antagonist (VKA) therapy,^22^ and the remaining trial focused on combined clopidogrel (75 mg/d) and aspirin (75–100 mg/d) versus warfarin.^21^ Patients with paroxysmal AF accounted for 11.4% to 32.8% of cases in these studies. The mean/median age ranged from 70 to 73 years, and females were less prevalent (30.8–41.5%). The mean CHADS_2_ (cardiac failure, hypertension, age, diabetes, stroke [doubled]) scores were approximately 2.0, with the exception of in the ROCKET-AF trial, in which it was 3.5. The median/mean follow-up periods were 1.1 to 2.0 years.^13,16,17,21,22,27–29,33–36^

**TABLE 1:**
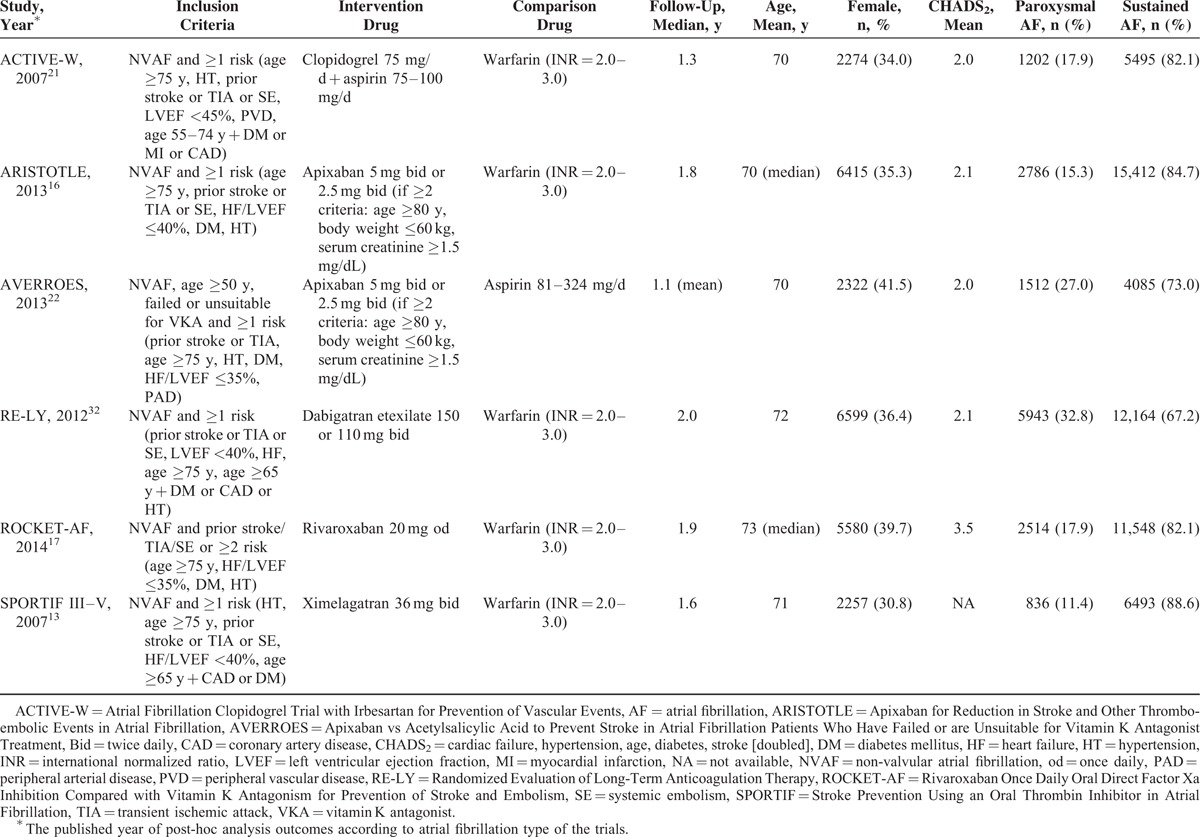
Study Characteristics

**FIGURE 2:**
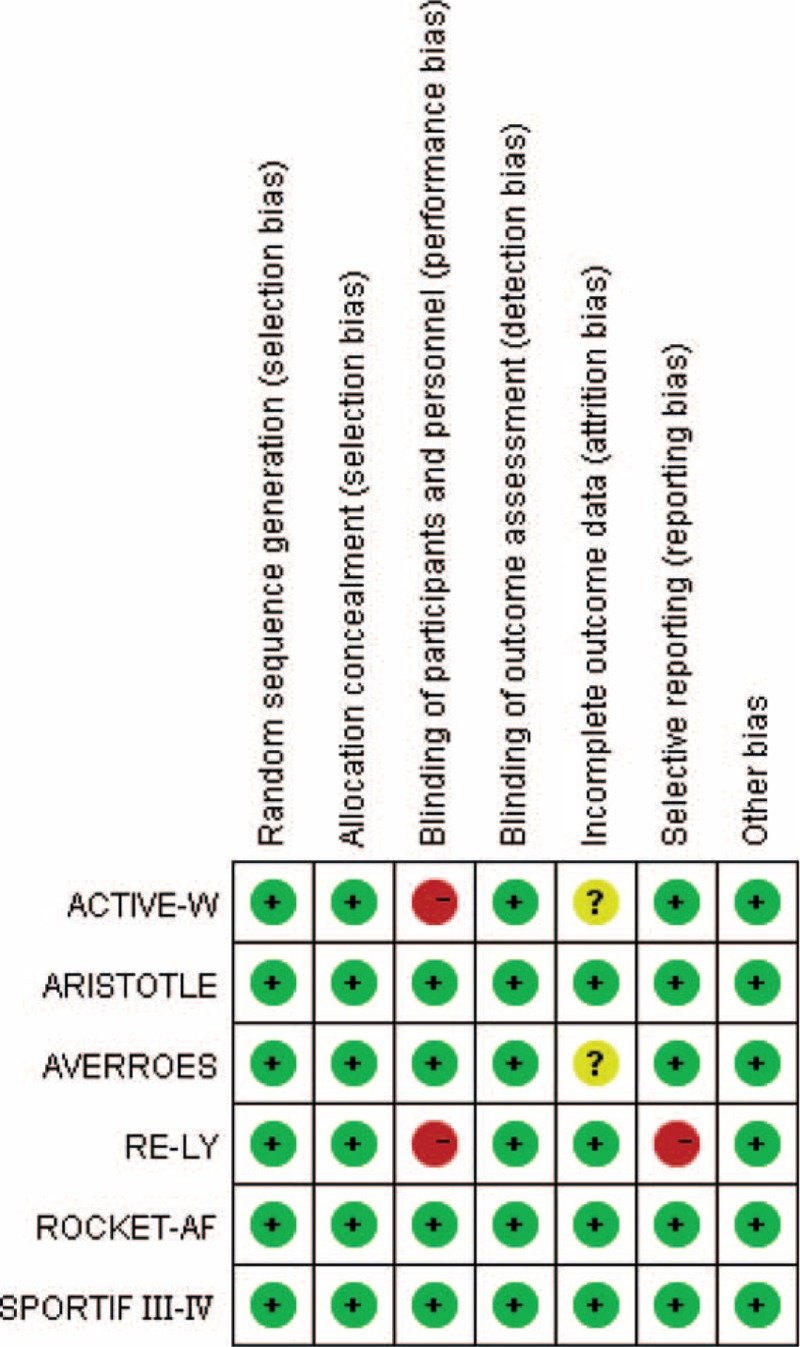
Risk of bias summary of the included studies.

For study quality assessment, 2 studies were rated as low risk, while 3 studies were evaluated as high risk according to the quality assessments scale for RCTs recommended by the Cochrane Collaboration.^31^ The Apixaban versus Acetylsalicylic Acid to Prevent Stroke in Atrial Fibrillation Patients who have Failed or are Unsuitable for Vitamin K Antagonist Treatment (AVERROES) trial^22^ did not provide information regarding dropouts (incomplete outcome data) and was thus rated as unclear risk (Fig. 2).

### Patient Characteristics

The baseline patient characteristics, stratified by AF type, are summarized in Table 2. A total of 69,990 AF patients with ≥1 risk factor for stroke were included; 14,793 (21.1%) had paroxysmal and 55,197 (78.9%) had sustained AF. The treatment assignment of anticoagulation (NOACs or warfarin) and antiplatelet agents were evenly distributed between patients with paroxysmal and sustained AF (*P* = 0.290). Compared to sustained AF, patients with paroxysmal AF were younger (≥75 years: 30.9% vs 37.7%; *P* < 0.001), more frequently female (41.3% vs 34.7%; *P* < 0.001), and less likely to have diabetes (27.4% vs 30.5%; *P* < 0.001) and cardiac dysfunction (34.6% vs 42.4%; *P* < 0.001). Higher rates of hypertension (88.1% vs 85.4%; *P* < 0.001) and previous stroke, transient ischemic attack, or systemic embolism (33.0% vs 29.6%; *P* < 0.001) were observed in paroxysmal AF. However, the CHADS_2_ score was balanced between patients with paroxysmal and sustained AF (score >2: 55.5% vs 54.8%; *P* = 0.340). Prior use of antithrombotic medications also differed, with lower VKA (69.0% vs 79.4%; *P* < 0.001) and higher aspirin (33.9% vs 26.6%; *P* < 0.001) use in paroxysmal AF patients.

**TABLE 2:**
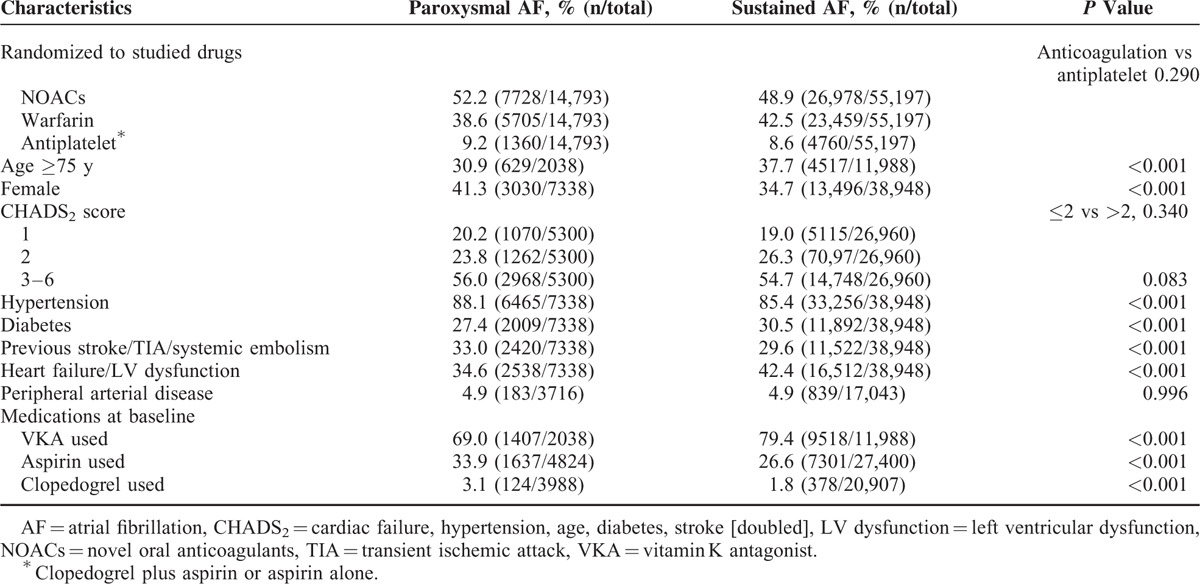
Baseline Characteristics of Patients With Paroxysmal and Sustained Atrial Fibrillation

### Outcomes

#### Outcomes by Atrial Fibrillation Type

**FIGURE 3:**
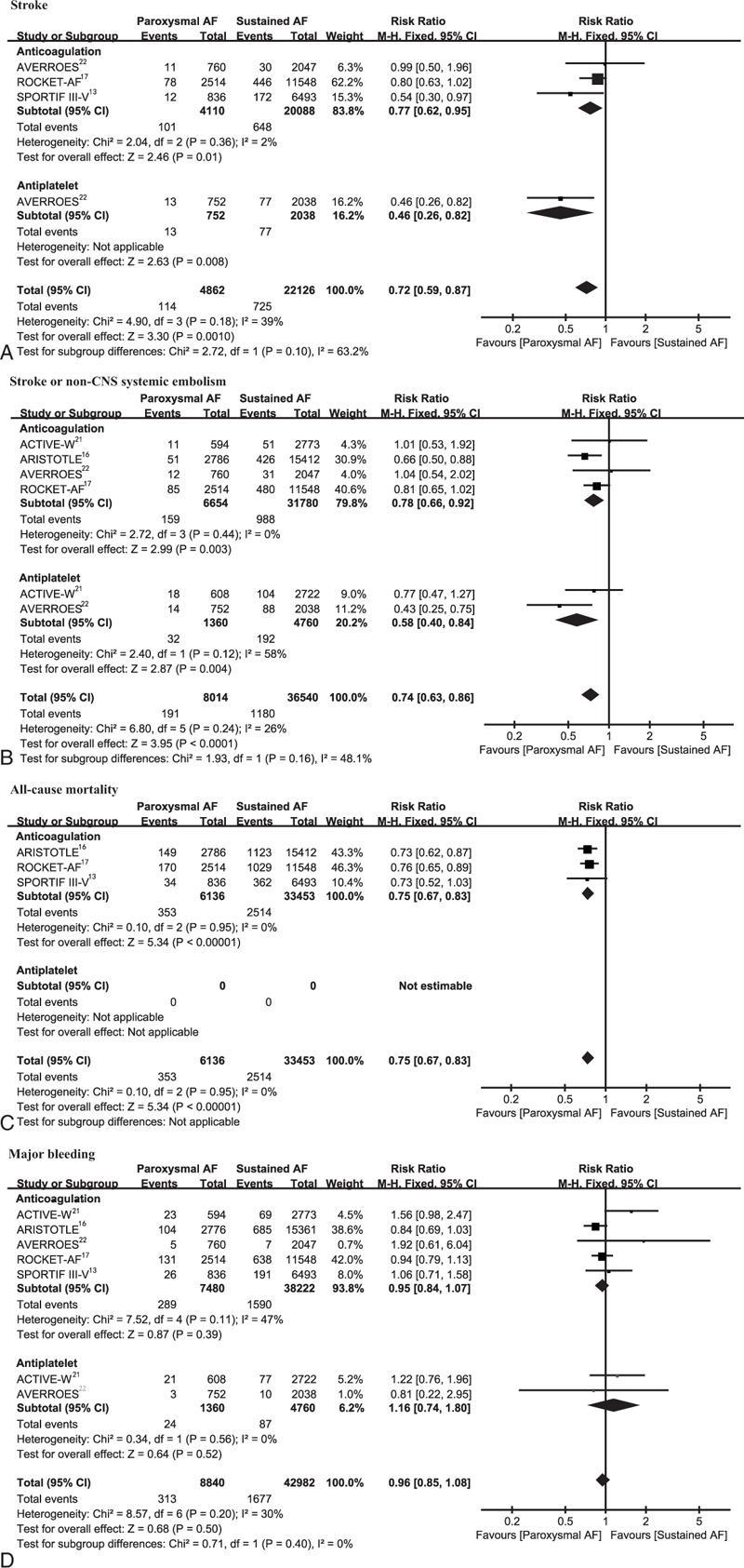
Efficacy (A–C) and safety (D) outcomes of paroxysmal versus sustained AF according to treatment of anticoagulation and antiplatelet. AF = atrial fibrillation, df = degrees of freedom, M–H = Mantel–Haenszel, non-CNS = non-central nervous system.

The outcomes stratified by AF type are shown in Figure 3. In patients receiving antithrombotic therapies, paroxysmal AF was associated with significantly lower risks of stroke (RR, 0.72; 95% CI, 0.59–0.87; *P* = 0.001), stroke or non-CNS systemic embolism (RR, 0.74; 95% CI, 0.63–0.86; *P* < 0.001), and all-cause mortality (RR, 0.75; 95% CI, 0.67–0.83; *P* < 0.001) as compared to sustained AF. The incidence of major bleeding was comparable between the 2 groups (RR, 0.96; 95% CI, 0.85–1.08; *P* = 0.50).

In the subgroup analysis, irrespective of anticoagulation or antiplatelet administration, paroxysmal AF patients consistently showed reduced stroke and stroke or non-CNS systemic embolism risks and similar major bleeding risk compared with sustained AF patients (Fig. 3). Likewise, in the anticoagulation treatment group, independent of whether NOACs or warfarin was administered, patients with paroxysmal AF showed favorable outcomes (see Figure 1, Supplemental Content, which illustrates the outcomes by AF type according to treatment with NOACs and warfarin). Specifically, despite statistical nonsignificance, patients with paroxysmal AF receiving NOACs tended to have reduced risks of stroke or systemic embolism (RR, 0.79; 95% CI, 0.62–1.01; *P* = 0.06).

#### Outcomes by Treatment

**FIGURE 4:**
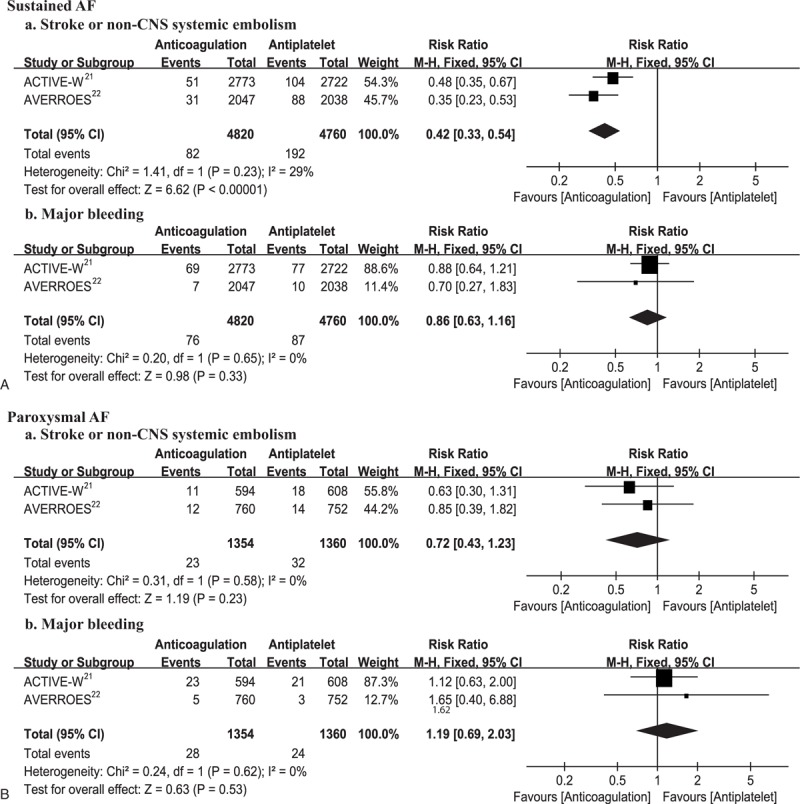
Efficacy (a) and safety (b) of anticoagulation versus antiplatelet according to AF type (A, B). AF = atrial fibrillation, df = degrees of freedom, M–H = Mantel–Haenszel, non-CNS = non-central nervous system.

The efficacy and safety of anticoagulation versus antiplatelet according to the AF type are shown in Figure 4. Anticoagulation treatment significantly reduced the risk of stroke or non-CNS systemic embolism in sustained AF patients (RR, 0.42; 95% CI, 0.33–0.54; *P* < 0.001), with no risk increase in major bleeding observed (RR, 0.86; 95% CI, 0.63–1.16; *P* = 0.33). For paroxysmal AF patients, we were not able to detect a significant difference between anticoagulation and antiplatelet treatment both for stroke or systemic embolism prevention (RR, 0.72; 95% CI, 0.43–1.23; *P* = 0.23) and major bleeding reduction (RR, 1.19; 95% CI, 0.69–2.03; *P* = 0.53).

**FIGURE 5:**
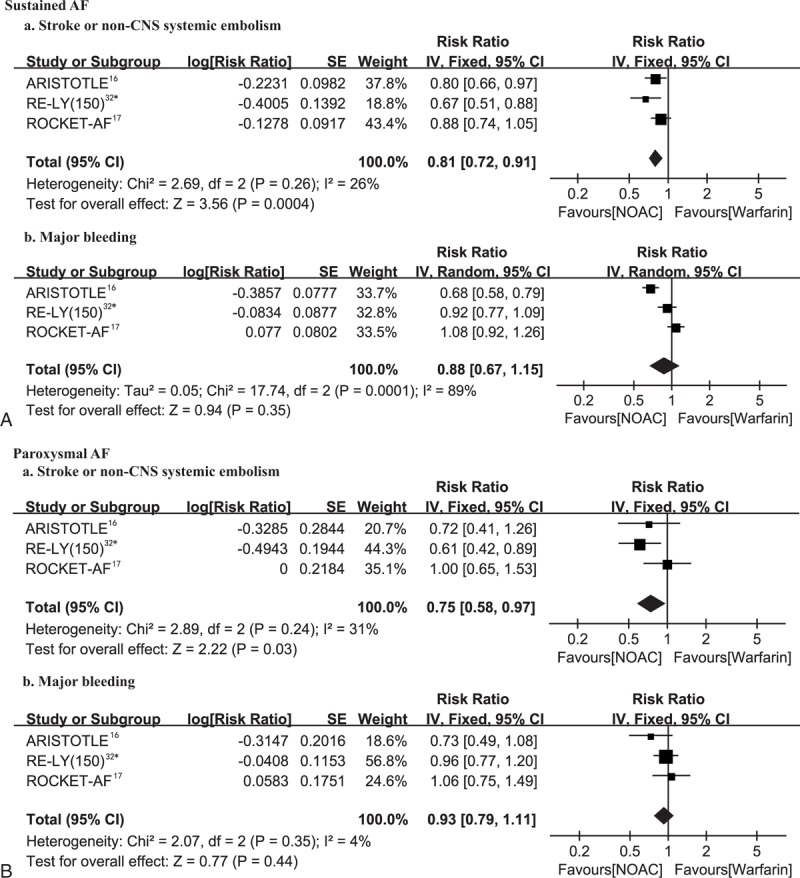
Efficacy (a) and safety (b) of NOACs versus warfarin according to AF type (A, B). AF = atrial fibrillation, df = degrees of freedom, IV = inverse variance, NAOCs = novel oral anticoagulants, SE = standard error. ^∗^Dabigatran 150 mg twice daily.

For anticoagulation (Fig. 5), NOACs (dabigatran etexilate 150 mg bid included) were more effective than warfarin for prevention of stroke or systemic embolism (sustained and paroxysmal: RR, 0.81; 95% CI, 0.72–0.91 and RR, 0.75; 95% CI, 0.58–0.97; respectively) and tended to show a lower risk of major bleeding (RR, 0.88; 95% CI, 0.67–1.15 and RR, 0.93; 95% CI, 0.79–1.11, respectively) irrespective of the AF type. Pooled analysis with another dose of dabigatran etexilate (110 mg bid) in the RE-LY trial as compared to warfarin showed similar results (see Figure 2, Supplemental Content, which illustrates the efficacy and safety of NOACs vs warfarin according to AF type).

## DISCUSSION

Our meta-analysis based on RCTs, incorporating 69,990 nonvalvular AF patients with ≥1 risk factor for stroke, has two main findings. First, post-antithrombotic therapy, paroxysmal AF patients had lower risks of stroke and composite of stroke or systemic embolism, better survival, and a comparable risk of major bleeding compared with sustained AF patients. Risk reductions of thromboembolic events in paroxysmal AF were consistently seen in patients receiving either anticoagulation (NOACs or warfarin) or antiplatelet agents. Second, compared with antiplatelets, anticoagulation therapy showed superior efficacy for prevention of stroke or systemic embolism in sustained AF, but we did not detect this superior efficacy or safety in paroxysmal AF.

Despite the serious consequences of embolic complications, it has not been well established whether the risk of embolization varies according to the type of AF. Several prior studies have revealed no difference in the outcomes between patients with paroxysmal and sustained AF.^11,19–21,23^ However, some studies exploring the problem under the setting of nonanticoagulation came to a different conclusion.^12,15^ A prospective cohort study showed that among paroxysmal AF patients not taking anticoagulants, the incidence of embolic complications increased from 2.0% to 5.1% per year after transition to chronic AF.^12^ The Loire Valley Atrial Fibrillation Project, a retrospective cohort study, also demonstrated that, in nonanticoagulated patients, the rate of stroke or thromboembolism was significantly higher in permanent AF.^15^ Our pooled data extended to show a greater thromboembolic risk in patients with more advanced forms of AF undergoing nonanticoagulation therapy in RCTs (the antiplatelet subgroup). However, pooled analysis of the Stroke Prevention in Atrial Fibrillation (SPAF) I-III trials, enrolling patients administered aspirin, suggested a comparable annual event rate between patients with intermittent (3.2%) and sustained AF (3.3%).^19^ Actually, the SPAF trials included AF patients not only assigned to aspirin but also a combination of aspirin plus inefficacious fixed-dose warfarin (international normalized ratio < 1.5), because the authors believed this would offer minimal additional protection against ischemic stroke.^37^ Moreover, unlike our population including only AF patients with ≥1 risk factor for stroke, 37.9% patients enrolled in the SPAF trials had no stroke risk factors.^19^

Besides those receiving antiplatelet, our study also consistently demonstrated thromboembolic risk reduction in patients with paroxysmal AF receiving anticoagulation. The differential intensity of anticoagulation use in patients with different AF types may have potentially contributed to the conflicting results. In the AVERROES trial, risk reduction of stroke or systemic embolism in paroxysmal AF patients, as compared to sustained AF, was found in aspirin-treated, but not apixaban-treated patients.^22^ Besides, the GISSI-AF trial and Euro Heart Survey did not reveal significant differences in the thromboembolic event rates between AF types; however, a substantially lower rate of anticoagulation was observed in patients with paroxysmal AF compared with sustained AF (26.5% vs 91.2% in GISSI-AF and 49.4% vs 77.9% in the Euro Heart Survey; *P* < 0.001).^20,23^ The confounding influence of anticoagulation may be attributable to its efficacy in thromboembolism prevention, especially for sustained AF, thus diminishing the power to distinguish risk differences between different AF forms.^18^ For this reason, we evaluated the outcomes separately in patients with paroxysmal and sustained AF in this meta-analysis. Although the ACTIVE W (Atrial Fibrillation Clopidogrel Trial with Irbesartan for Prevention of Vascular Events) trial showed a similar risk of thromboembolic events for both types of arrhythmia irrespective of treatment with anticoagulation or antiplatelets, it enrolled quite a limited number of AF patients (n = 6697) compared with our study (n = 69,990).

Previous studies have observed different risk factor profiles according to the type of AF, and concluded that the different outcomes were due to these different risk factors, such as increasing age.^19,21,38^ The ROCKET-AF trial, including consistently anticoagulated patients, with well-balanced CHADS_2_ scores at baseline and treatment assignment between paroxysmal and sustained AF patients, still demonstrated lower embolic events and better survival in paroxysmal AF patients.^17^ In our analysis, paroxysmal AF patients were younger and had less heart dysfunction, which indicated that this subgroup was at an early stage of arrhythmia; however, they were associated with higher rates of hypertension and previous thromboembolic events, and the rates of CHADS_2_ score >2 were equivalent between the AF types (55.5% vs 54.8%; *P* = 0.340). Moreover, antithrombotic prophylactic VKA use before entry was less frequent in patients with paroxysmal AF (69.0% vs 79.4%; *P* < 0.001). Our data provide support for a greater risk related to the more advanced form of AF and suggest that the worse outcomes in advanced AF could be attributed not only to stroke risk factors but may also be associated with the hemodynamic disorders resulting from electromechanical disturbances of the rhythm.

The current guidelines, based mainly on the results of the SPAF trials, which showed no difference in stroke risk between intermittent and sustained AF,^19^ recommend similar antithrombotic strategies for AF patients based on risk stratification of the CHA_2_DS_2_-VASc score, without considering the AF type.^5^ However, our pooled result of RCTs demonstrated that the AF type is a significant predictor for thromboembolism, and it might hence be helpful in risk stratification or for improvement of risk prediction if combined with other risk factors in the current risk prediction models.

Paroxysmal AF has not received as much attention as sustained AF, mainly due to its lack of symptoms and difficulty of detection.^7^ Recent efforts have been made to improve its detection, including advances in prolonged Holter (24 hours to 7 days) monitoring, automatic or patient-activated event loop recorders, and insertable cardiac monitors.^7–9,40–44^ Nonetheless, despite these diagnostic improvements, few studies have specifically focused on the comparison of antithrombotic therapy for paroxysmal AF. Although dose-adjusted warfarin, compared with aspirin, has been demonstrated to significantly decrease stroke and cardiovascular events independent of the AF type in a prior meta-analysis in 2002,^45^ the appearance of novel antiplatelet agents (eg, clopidogrel) and NOACs have resulted in the choice of antithrombotic prophylaxis for paroxysmal AF patients becoming more complicated. Herein, we were unable to detect significant difference in the efficacy or safety between anticoagulation and antiplatelet therapy for paroxysmal AF, although anticoagulation showed favorable efficacy for sustained AF. Of note, our pooled data of the anticoagulation and antiplatelet comparison included only 2 trials (ACTIVE W and AVERROES)^21,22^ with 2714 patients having paroxysmal AF, in which 1 used warfarin and 1 used a NOAC (apixaban) for anticoagulation, and 1 used a combination of aspirin plus clopidogrel and 1 used aspirin alone for antiplatelet treatment. The combination of apixaban with warfarin, and aspirin plus clopidogrel with aspirin alone in our analysis might be considered unreasonable, but post-hoc analysis of the ARISTOTLE trial^16^ suggested that for the paroxysmal AF patients, apixaban was not superior to warfarin either for stroke prevention or major bleeding reduction and also the ACTIVE A trial^46^ showed a similar effect of aspirin with clopidogrel and aspirin alone for stroke prevention in this AF group. Besides our pooled result showing no difference, sensitive analysis of the ACTIVE W^21^ or the AVERROES trial^22^ 1 consistently did not reveal the superiority of warfarin or apixaban over aspirin plus clopidogrel or aspirin for paroxysmal AF (both 95% CIs crossed 1). The low event rate of the study outcome may have been due to appropriate management of the associated stroke risk factors under supervision in these large clinical trials. Paroxysmal AF is at significant risk of stroke, relative to patients without AF,^39^ and our data do not support withholding anticoagulation in these patients. Further, our result of the nonsignificant difference between anticoagulation and antiplatelet agents in paroxysmal AF may be controversial, but we consider that the optimal antithrombotic strategy for this AF form awaits investigation. We call for the ongoing or coming trials of the antithrombotic drugs for AF to further compare their effect and safety with regard to AF type.

As paroxysmal AF has been suggested to be a potential cause for patients with cryptogenic ischemic stroke^7,8^ or ESUS,^6^ implementing optimal antithrombotic prophylaxis is essential for secondary stroke prevention. Our study mainly showed the treatment effect for primary, rather than secondary stroke prevention, since only 33.3% and 29.6% of paroxysmal and sustained AF patients, respectively, had a previous history of stroke or transient ischemic attack or systemic embolism. Thus, the optimal treatment choice for secondary stroke prevention in patients with paroxysmal AF remains unknown. The outcomes of 2 ongoing large trials (Dabigatran Etexilate for Secondary Stroke Prevention in Patients With Embolic Stroke of Undetermined Source [RE-SPECT ESUS, NCT02239120] and Rivaroxaban Versus Aspirin in Secondary Prevention of Stroke and Prevention of Systemic Embolism in Patients With Recent Embolic Stroke of Undetermined Source [NAVIGATE ESUS, NCT02313909]), investigating the efficacy and safety of dabigatran etexilate and rivaroxaban with aspirin in patients recently diagnosed as having ESUS, may help provide insight into the effects of anticoagulation (NOACs) and antiplatelet (aspirin) therapy for paroxysmal AF and provide guidance in the antithrombotic choice.

## LIMITATIONS

There are several limitations to the present study. We could not provide clear conclusion of the superiority of anticoagulation or antiplatelets for paroxysmal AF, as there were far few patients with paroxysmal AF in the 2 included studies, and the event rate was low. Moreover, because we did not have access to the individual patient data for the included trials, our statistical analysis was performed at the study level, resulting in some incompleteness in the baseline characteristics and outcome assessment data. Several baseline characteristics were significantly different between the paroxysmal and sustained AF patients, despite the CHADS_2_ score being evenly distributed; however, multivariate analysis of the associations between risk factors and outcomes and sub-analysis of paroxysmal AF according to risk score cannot be performed in study-level analyses. The CHA_2_DS_2_-VASc score is preferred to the CHADS_2_ score for stroke risk stratification of AF, but the difference of CHADS_2_ score rather than the CHA_2_DS_2_-VASc was assessed herein, as the CHA_2_DS_2_-VASc score was not widely used during the ongoing period of these RCTs. Besides, there were 4 agents of NOACs and 2 different antiplatelet regimens (Aspirin 81–324 mg/d; Clopidogrel 75 mg/d + aspirin 75–100 mg/d) among the included trials, so that clinical heterogeneity should be taken into consideration for the pooled result. Lastly, while the type of AF was determined at the time of enrollment by the local investigators according to at least 2 documented ECGs and previous medical history, the burden of paroxysmal AF is heterogeneous and it may progress to persistent or permanent AF during follow-up. The incidence of embolic complications has been reported to greatly rise (from 2.7% to 13.3%) during the first year after paroxysmal transition to sustained AF^12^; however, in an intention-to-treat analysis, this would only strengthen our finding that paroxysmal AF patients carry a lower thromboembolic risk compared with sustained AF patients, as the increased events were calculated in the paroxysmal AF group.

## CONCLUSIONS

In conclusion, among non-valvular AF patients with ≥1 risk factor for stroke receiving either anticoagulation (NOACs or warfarin) or antiplatelet agents, paroxysmal AF patients consistently showed a reduced risk of stroke or systemic embolism and comparable risk of major bleeding as compared with sustained AF patients. The AF type might be helpful in risk stratification for antithrombotic prophylaxis determination. Anticoagulation, especially NOACs, may represent the optimal antithrombotic choice for sustained AF. However, for those with paroxysmal AF, the best therapeutic strategy between diversified anticoagulant or antiplatelet agents awaits further confirmation.

## Supplementary Material

Supplemental Digital Content
