# Supplementary material for: Stroke Event Rates and the Optimal Antithrombotic Choice of Patients With Paroxysmal Atrial Fibrillation: A Systematic Review and Meta-Analysis of Randomized Controlled Trials
Source: Medicine (Baltimore). 2015 Dec 31;94(52):e2364. doi: 10.1097/MD.0000000000002364 (PMC5291617; doi:10.1097/MD.0000000000002364)
Supplement: Supplemental Digital Content [file medi-94-e2364-s001.pdf]

Supplemental content

TEXT 1: Search strategy

(1) Cochrane Central Register of Controlled Trials (Ovid)

1. exp *Atrial Fibrillation/ (Map Term to Subject Heading)
2. (atrial fibrillation or AF or atrium fibrillation or auricular fibrillation).tw.
3. 1 or 2
4. exp *Stroke/ (Map Term to Subject Heading)
5. (stroke or brain infarction or brain vascular accident or cerebrovascular accident).tw.
6. 4 or 5
7. exp *Embolism/ (Map Term to Subject Heading)
8. (embolism or embolus).tw.
9. 7 or 8
10. 3 and 6 and 9

(2) MEDLINE (Ovid)

1. Randomized controlled trials as Topic/
2. Randomized controlled trial/
3. Random allocation/
4. Double blind method/
5. Single blind method/
6. Clinical trial/
7. exp Clinical Trials as Topic/
8. or/1-7
9. (clinic$ adj trial$1).tw.
10. ((singl$ or doubl$ or treb$ or tripl$) adj (blind$3 or mask$3)).tw.
11. Placebos/
12. Placebo$.tw.
13. Randomly allocated.tw.
14. (allocated adj2 random).tw.
15. or/9-14
16. 8 or 15
17. Case report.tw.
18. Letter/
19. Historical article/
20. Review of reported cases.pt.

21. Review, multicase.pt.
22. or/17-21
23. 16 not 22
24. exp *Atrial Fibrillation/
25. (atrial fibrillation or atrium fibrillation or auricular fibrillation or AF).tw.
26. 24 or 25
27. exp *Stroke/
28. (stroke or brain infarction or brain vascular accident or cerebrovascular accident).tw.
29. 27 or 28
30. exp *Embolism/
31. (embolism or embolus).tw.
32. 30 or 31
33. 23 and 26 and 29 and 32

(3) EMBASE (Ovid)
1. Clinical trial/
2. Randomized controlled trial/
3. Randomization/
4. Single blind procedure/
5. Double blind procedure/
6. Crossover procedure/
7. Placebo/
8. Randomi?ed controlled trial$.tw.
9. Rct.tw.
10. Random allocation.tw.
11. Randomly allocated.tw.
12. Allocated randomly.tw.
13. (allocated adj2 random).tw.
14. Single blind$.tw.
15. Double blind$.tw.
16. ((treble or triple) adj blind$).tw.
17. Placebo$.tw.
18. Prospective study/
19. or/1-18
20. Case study/
21. Case report.tw.
22. Abstract report/ or letter/
23. or/20-22

24. 19 not 23
25. exp *heart atrium fibrillation/
26. (atrial fibrillation or atrium fibrillation or auricular fibrillation or AF).tw.
27. 25 or 26
28. exp *cerebrovascular accident/
29. (stroke or brain infarction or brain vascular accident or cerebrovascular accident).tw.
30. 28 or 29
31. exp *embolism/
32. (embolism or embolus).tw.
33. 31 or 32
34. 24 and 27 and 30 and 33

TEXT 2: Definition and classification of AF in the included studies

1. ACTIVE-W[21]

Patients were classified as having paroxysmal, persistent, or permanent AF by local investigators. Patients with permanent AF had electrocardiogram-documented AF at the time of enrollment and no evidence of SR in the 6 months before randomization. Patients with paroxysmal or persistent AF may not have been in AF at the time of randomization, but had electrocardiogram-documented AF on 2 separate occasions, at least 2 weeks apart, in the 6 months before randomization.

2. ARISTOTLE[16]

The definitions of paroxysmal, persistent, and permanent AF used in the trial were in accordance with the definitions used in the 2006 American College of Cardiology/American Heart Association/European Society of Cardiology AF guidelines. Specifically, paroxysmal AF was defined as recurrent AF that terminates spontaneously, persistent AF was defined as AF that is sustained beyond 7 days, and permanent AF was defined as long-standing AF in which restoring and/or maintaining sinus rhythm has failed or has been foregone. The prior need for a cardioversion did not automatically lead to classifying AF as persistent; the subsequent occurrence of self-terminating paroxysms of AF in such patients led to classifying them as having paroxysmal AF. The type of AF was classified by the sites at randomization when they had to specify whether a particular patient had paroxysmal or persistent/permanent (combined into one category) AF or atrial flutter documented by an ECG at the time of enrolment or whether a patient had AF or atrial flutter documented on two separate occasions at least 2 weeks apart in the 6 months prior to enrolment.

3. AVERROES[22]

Patients had AF documented by 12-lead electrocardiography (ECG) on the day of screening. If not in AF on the day of screening, AF had to be documented in the 6 months prior to enrolment by 12-lead ECG or as an episode at least 5 min in duration on a rhythm strip or Holter recording. Pacemaker or ICD electrogram recordings could be used to document AF but the duration of AF had to be at least 30 min if this was the only documentation of AF.

Patients were classified as having paroxysmal, persistent or permanent AF at baseline by local investigators. Patients with permanent AF had ECG documented AF at the time of enrolment and no evidence of sinus rhythm (SR) in the 6 months prior to randomization. Patients with paroxysmal or persistent AF may not have been in AF at

the time of randomization, but had ECG documented AF in the 6 months prior to randomization.

4. RE-LY[32]

AF is documented by ECG on the day of screening or randomization. The patient has had a symptomatic episode of paroxysmal or persistent AF documented by 12-lead ECG within 6 months before randomization. There is documentation of symptomatic or asymptomatic paroxysmal or persistent AF on 2 separate occasions, at least 1 day apart, one of which is within 6 months before randomization. In this case, AF may be documented by 12 lead ECG, rhythm strip, pacemaker/ICD electrogram, or Holter ECG. The duration of AF should be at least 30 s. Electrograms (not marker channels or mode switch episodes) from pacemakers and defibrillators can be used to document only 1 episode of paroxysmal or persistent AF.

5. ROCKET-AF[17]

All the patients had to have electrocardiographic evidence of AF within 30 days prior to randomization; additionally, they had to have medical evidence of AF within the previous year. Patients were categorized by the enrolling physician at baseline as having either paroxysmal (lasting ≤7 days at any time) or persistent AF (>7 days at a time); no other AF types were provided as choices.

6. SPORTIF III-V[13]

AF was defined as paroxysmal or persistent nonvalvular AF verified by at least two ECG recordings, one of which was made within 2 weeks before randomization. The “persistent (constant)” AF was made by the study investigator prior to randomization based on baseline ECG recordings and prior clinical history. Thus, those categorized as “persistent (constant)” AF would include those with persistent or permanent AF. Whilst the categorization depended upon investigator classification, the diagnosis would have been verified by the trial monitors review of source documents.

A.Stroke or non-CNS systemic embolism

Study or Subgroup	Paroxysmal AF Events	Paroxysmal AF Total	Sustained AF Events	Sustained AF Total	Weight	Risk Ratio M-H, Fixed, 95% CI
**NOACs**						
ARISTOTLE[16]	21	1374	191	7744	37.4%	0.62 [0.40, 0.97]
AVERROES[22]	12	760	31	2047	10.9%	1.04 [0.54, 2.02]
ROCKET-AF[17]	42	1245	225	5786	51.7%	0.87 [0.63, 1.20]
**Subtotal (95% CI)**		**3379**		**15577**	**100.0%**	**0.79 [0.62, 1.01]**
Total events	75		447			
Heterogeneity: Chi² = 2.12, df = 2 (P = 0.35); I² = 6%						
Test for overall effect: Z = 1.86 (P = 0.06)						
**Warfarin**						
ACTIVE-W[21]	11	594	51	2773	9.8%	1.01 [0.53, 1.92]
ARISTOTLE[16]	30	1412	235	7668	39.9%	0.69 [0.48, 1.01]
ROCKET-AF[17]	43	1269	255	5762	50.3%	0.77 [0.56, 1.05]
**Subtotal (95% CI)**		**3275**		**16203**	**100.0%**	**0.76 [0.61, 0.95]**
Total events	84		541			
Heterogeneity: Chi² = 0.96, df = 2 (P = 0.62); I² = 0%						
Test for overall effect: Z = 2.37 (P = 0.02)						

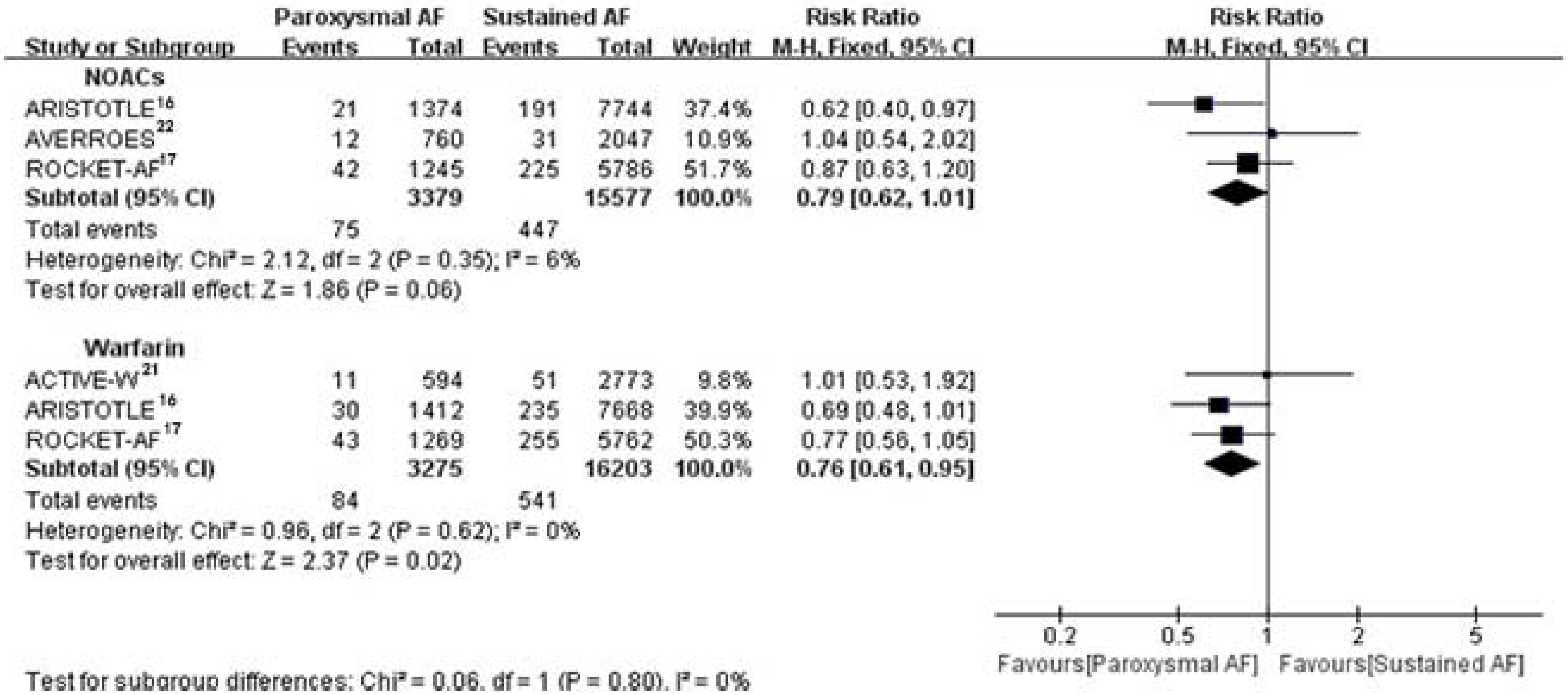

Test for subgroup differences: Chi² = 0.06, df = 1 (P = 0.80), I² = 0%

B. Major bleeding

Study or Subgroup	Paroxysmal AF Events	Paroxysmal AF Total	Sustained AF Events	Sustained AF Total	Weight	Risk Ratio M-H, Random, 95% CI
**NOACs**						
ARISTOTLE[16]	44	1374	283	7744	39.3%	0.88 [0.64, 1.20]
AVERROES[22]	5	760	7	2047	2.9%	1.92 [0.61, 6.04]
ROCKET-AF[17]	66	1245	323	5786	57.8%	0.95 [0.73, 1.23]
**Subtotal (95% CI)**		**3379**		**15577**	**100.0%**	**0.94 [0.77, 1.14]**
Total events	115		613			
Heterogeneity: Tau² = 0.00; Chi² = 1.70, df = 2 (P = 0.43); I² = 0%						
Test for overall effect: Z = 0.63 (P = 0.53)						
**Warfarin**						
ACTIVE-W[21]	23	594	69	2773	24.1%	1.56 [0.98, 2.47]
ARISTOTLE[16]	60	1412	402	7668	37.8%	0.81 [0.62, 1.06]
ROCKET-AF[17]	65	1269	315	5762	38.2%	0.94 [0.72, 1.22]
**Subtotal (95% CI)**		**3275**		**16203**	**100.0%**	**1.00 [0.74, 1.37]**
Total events	148		786			
Heterogeneity: Tau² = 0.05; Chi² = 5.76, df = 2 (P = 0.06); I² = 65%						
Test for overall effect: Z = 0.01 (P = 0.99)						

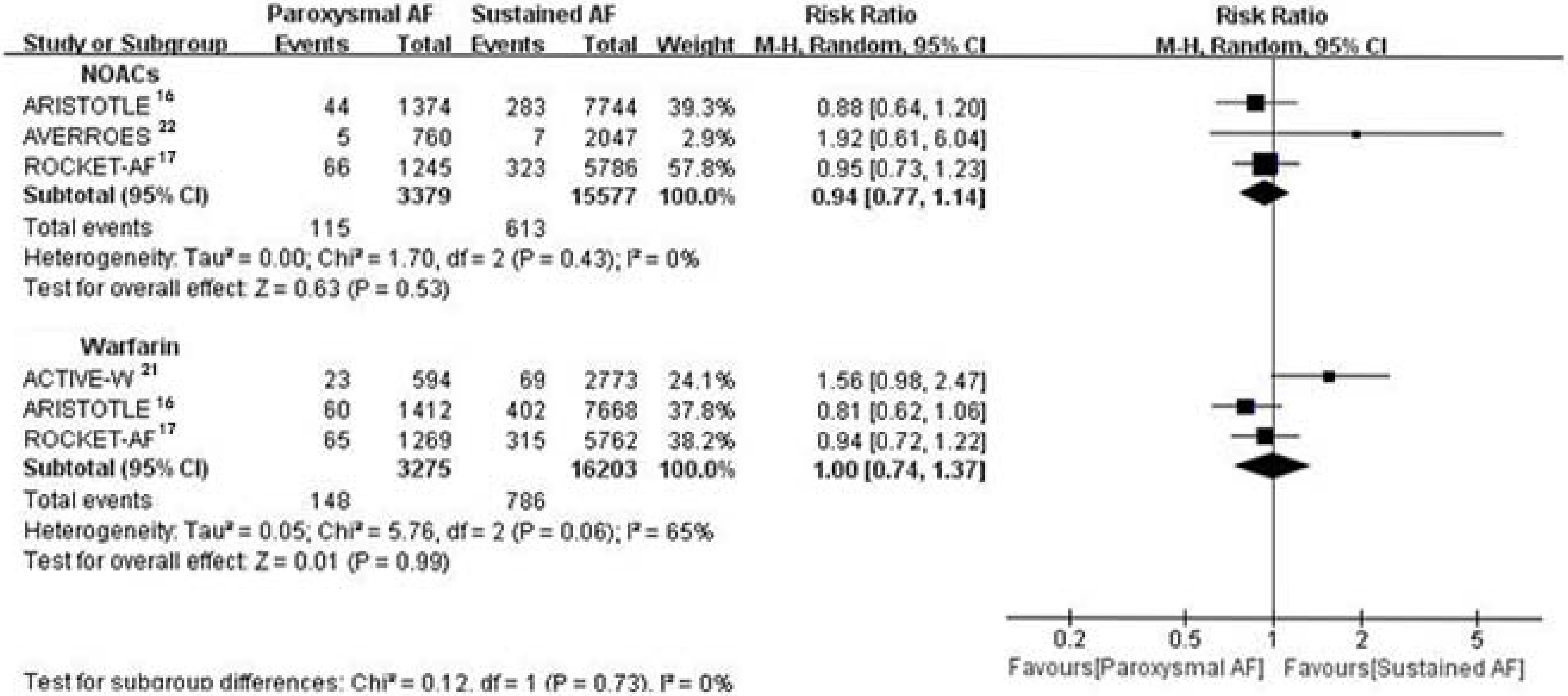

Test for subgroup differences: Chi² = 0.12, df = 1 (P = 0.73), I² = 0%

FIGURE 1. Efficacy (A) and safety (B) outcomes of paroxysmal vs. sustained AF according to treatments of NOACs and warfarin. AF=atrial fibrillation, df=degrees of freedom, M-H=Mantel-Haenszel, NOACs=novel oral anticoagulants.

A. Sustained AF

a. Stroke or non-CNS systemic embolism

Study or Subgroup	log[Risk Ratio]	SE	Weight	Risk Ratio IV, Fixed, 95% CI
ARISTOTLE[16]	-0.2231	0.0982	35.7%	0.80 [0.66, 0.97]
RE-LY(110)[32*]	0.0392	0.1214	23.4%	1.04 [0.82, 1.32]
ROCKET-AF[17]	-0.1278	0.0917	40.9%	0.88 [0.74, 1.05]
**Total (95% CI)**			**100.0%**	**0.88 [0.79, 0.99]**

Heterogeneity: $Chi^2 = 2.83$, df = 2 (P = 0.24); $I^2 = 29\%$
Test for overall effect: Z = 2.09 (P = 0.04)

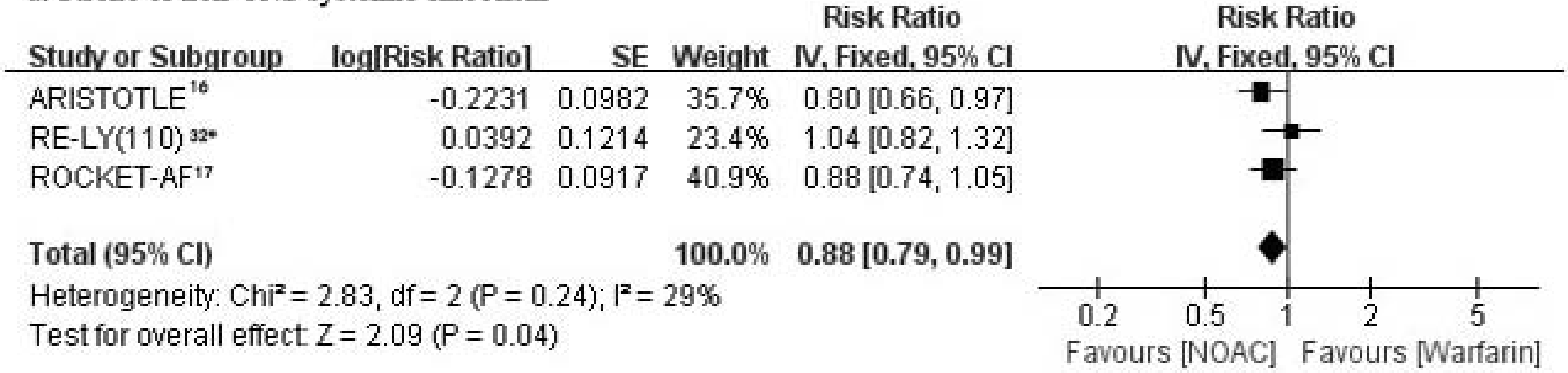

b. Major bleeding

Study or Subgroup	log[Risk Ratio]	SE	Weight	Risk Ratio IV, Random, 95% CI
ARISTOTLE[16]	-0.3857	0.0777	33.8%	0.68 [0.58, 0.79]
RE-LY(110)[32*]	-0.1985	0.0895	32.7%	0.82 [0.69, 0.98]
ROCKET-AF[17]	0.077	0.0802	33.6%	1.08 [0.92, 1.26]
**Total (95% CI)**			**100.0%**	**0.84 [0.64, 1.11]**

Heterogeneity: $Tau^2 = 0.05$; $Chi^2 = 17.29$, df = 2 (P = 0.0002); $I^2 = 88\%$
Test for overall effect: Z = 1.21 (P = 0.23)

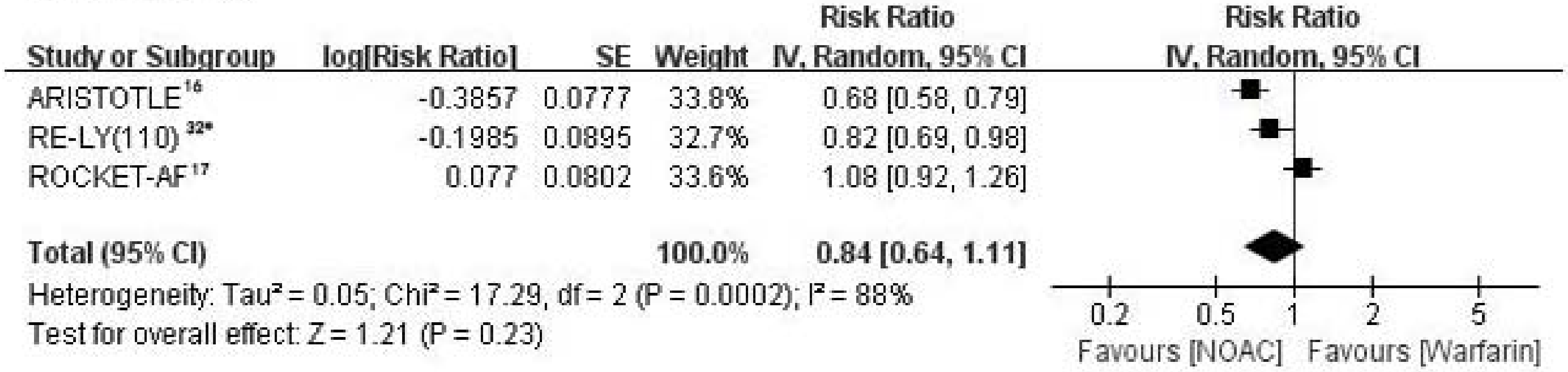

B. Paroxysal AF

a. Stroke or non-CNS systemic embolism

Study or Subgroup	log[Risk Ratio]	SE	Weight	Risk Ratio IV, Fixed, 95% CI
ARISTOTLE[16]	-0.3285	0.2844	21.0%	0.72 [0.41, 1.26]
RE-LY(110)[32*]	-0.5108	0.1977	43.4%	0.60 [0.41, 0.88]
ROCKET-AF[17]	0	0.2184	35.6%	1.00 [0.65, 1.53]
**Total (95% CI)**			**100.0%**	**0.75 [0.58, 0.97]**

Heterogeneity: $Chi^2 = 3.03$, df = 2 (P = 0.22); $I^2 = 34\%$
Test for overall effect: Z = 2.23 (P = 0.03)

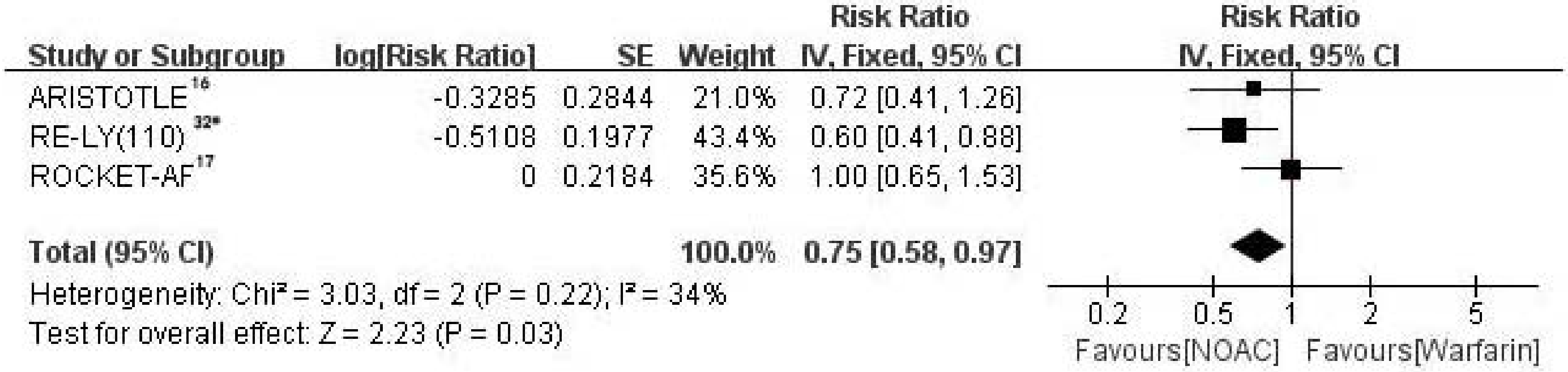

b. Major bleeding

Study or Subgroup	log[Risk Ratio]	SE	Weight	Risk Ratio IV, Fixed, 95% CI
ARISTOTLE[16]	-0.3147	0.2016	20.0%	0.73 [0.49, 1.08]
RE-LY(110)[32*]	-0.2485	0.1235	53.4%	0.78 [0.61, 0.99]
ROCKET-AF[17]	0.0583	0.1751	26.6%	1.06 [0.75, 1.49]
**Total (95% CI)**			**100.0%**	**0.84 [0.70, 1.00]**

Heterogeneity: $Chi^2 = 2.61$, df = 2 (P = 0.27); $I^2 = 23\%$
Test for overall effect: Z = 2.00 (P = 0.05)

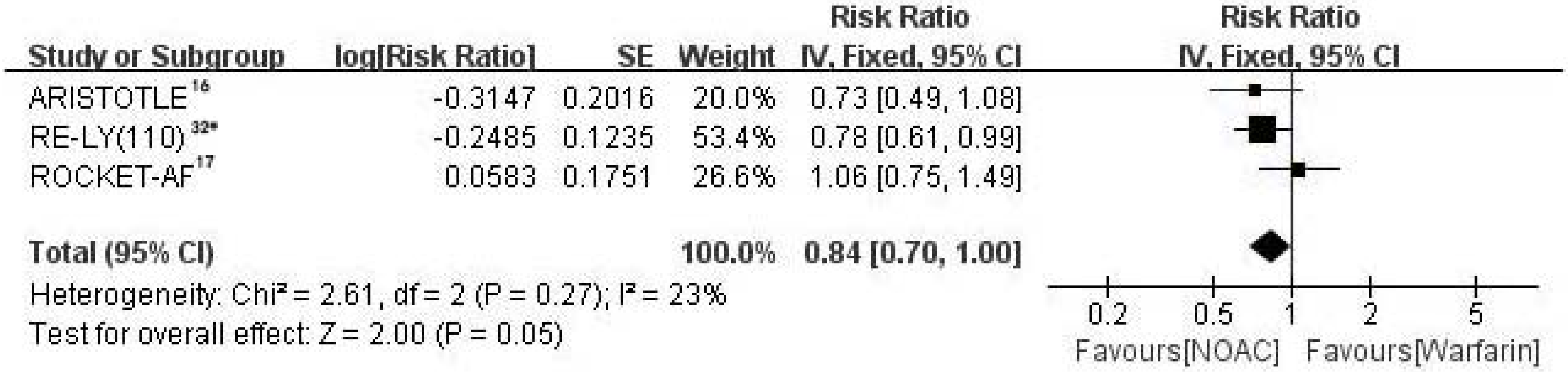

FIGURE 2. Efficacy (a) and safety (b) of NOAC vs. warfarin according to AF type (A, B). AF=atrial fibrillation, Bid=twice daily, DE=dabigatran etexilate, df=degrees of freedom, IV=Inverse Variance, NOAC=novel oral anticoagulant, SE=standard error.

***Dabigatran 110mg twice daily.**
